# Comparison of SARS-CoV-2 Detection by Rapid Antigen and by Three Commercial RT-qPCR Tests: A Study from Martin University Hospital in Slovakia

**DOI:** 10.3390/ijerph18137037

**Published:** 2021-07-01

**Authors:** Zuzana Dankova, Elena Novakova, Maria Skerenova, Veronika Holubekova, Vincent Lucansky, Dana Dvorska, Dusan Brany, Zuzana Kolkova, Jan Strnadel, Sandra Mersakova, Katarina Janikova, Marek Samec, Michal Pokusa, Martin Petras, Miroslava Sarlinova, Ivana Kasubova, Dusan Loderer, Vladimira Sadlonova, Jana Kompanikova, Nina Kotlebova, Adriana Kompanikova, Martina Hrnciarova, Andrea Stanclova, Martina Antosova, Anton Dzian, Vladimir Nosal, Ivan Kocan, Dalibor Murgas, Dusan Krkoska, Andrea Calkovska, Erika Halasova

**Affiliations:** 1 Biomedical Center Martin, Jessenius Faculty of Medicine in Martin, Comenius University in Bratislava, 036 01 Martin, Slovakia; zuzana.dankova@uniba.sk (Z.D.); maria.skerenova@uniba.sk (M.S.); veronika.holubekova@uniba.sk (V.H.); vincent.lucansky@uniba.sk (V.L.); dana.dvorska@uniba.sk (D.D.); dusan.brany@uniba.sk (D.B.); zuzana.snahnicanova@uniba.sk (Z.K.); jan.strnadel@uniba.sk (J.S.); sandra.mersakova@uniba.sk (S.M.); chovancova64@uniba.sk (K.J.); marek.samec@uniba.sk (M.S.); michal.pokusa@uniba.sk (M.P.); martin.petras@uniba.sk (M.P.); miroslava.sarlinova@uniba.sk (M.S.); ivana.kasubova@uniba.sk (I.K.); dusan.loderer@uniba.sk (D.L.); erika.halasova@uniba.sk (E.H.); 2 Department of Microbiology and Immunology, Jessenius Faculty of Medicine in Martin, Comenius University in Bratislava, 036 01 Martin, Slovakia; vladimira.sadlonova@uniba.sk (V.S.); jana.kompanikova@uniba.sk (J.K.); nina.kotlebova@uniba.sk (N.K.); 3 Department of Pathological Anatomy, Jessenius Faculty of Medicine in Martin, Comenius University in Bratislava, 036 01 Martin, Slovakia; avoj@centrum.sk; 4 Clinic of Obstetrics and Gynecology, Jessenius Faculty of Medicine in Martin, Comenius University in Bratislava, 036 01 Martin, Slovakia; 5 Hospital Hygiene Department, Martin University Hospital, 036 01 Martin, Slovakia; kompanikova@unm.sk (A.K.); mhrnciarova@unm.sk (M.H.); 6 Modern Medical Leadership, s.r.o., 036 01 Martin, Slovakia; matveja@gmail.com; 7 Department of Thoracic Surgery, Martin University Hospital, Jessenius Faculty of Medicine in Martin, Comenius University in Bratislava, 036 01 Martin, Slovakia; anton.dzian@uniba.sk; 8 Clinic of Neurology, Martin University Hospital, Jessenius Faculty of Medicine in Martin, Comenius University in Bratislava, 036 01 Martin, Slovakia; vladimir.nosal@uniba.sk; 9 Clinic of Pneumology and Phthisiology, Martin University Hospital, Jessenius Faculty of Medicine in Martin, Comenius University in Bratislava, 036 01 Martin, Slovakia; ivan.kocan@uniba.sk; 10 Department of Pediatric Surgery, Martin University Hospital, Jessenius Faculty of Medicine in Martin, Comenius University in Bratislava, 036 01 Martin, Slovakia; dalibor.murgas@uniba.sk; 11 Department of Infectious Diseases and Travel Medicine, Martin University Hospital, Jessenius Faculty of Medicine in Martin, Comenius University in Bratislava, 036 01 Martin, Slovakia; krkoska@unm.sk; 12 Department of Physiology, Jessenius Faculty of Medicine in Martin, Comenius University in Bratislava, 036 01 Martin, Slovakia; andrea.calkovska@uniba.sk

**Keywords:** SARS-CoV-2, antigen, RT-qPCR, Ct, sensitivity, specificity, NPV—negative predictive value, PPV—positive predictive value

## Abstract

The global pandemic of coronavirus disease 2019 (COVID-19) caused by the severe acute respiratory syndrome coronavirus 2 (SARS-CoV-2) is having a tremendous impact on the global economy, health care systems and the lives of almost all people in the world. The Central European country of Slovakia reached one of the highest daily mortality rates per 100,000 inhabitants in the first 3 months of 2021, despite implementing strong prophylactic measures, lockdowns and repeated nationwide antigen testing. The present study reports a comparison of the performance of the Standard Q COVID-19 antigen test (SD Biosensor) with three commercial RT-qPCR kits (vDetect COVID-19-MultiplexDX, gb SARS-CoV-2 Multiplex-GENERI BIOTECH Ltd. and Genvinset COVID-19 [E]-BDR Diagnostics) in the detection of infected individuals among employees of the Martin University Hospital in Slovakia. Health care providers, such as doctors and nurses, are classified as “critical infrastructure”, and there is no doubt about the huge impact that incorrect results could have on patients. Out of 1231 samples, 14 were evaluated as positive for SARS-CoV-2 antigen presence, and all of them were confirmed by RT-qPCR kit 1 and kit 2. As another 26 samples had a signal in the *E* gene, these 40 samples were re-isolated and subsequently re-analysed using the three kits, which detected the virus in 22, 23 and 12 cases, respectively. The results point to a divergence not only between antigen and RT-qPCR tests, but also within the “gold standard” RT-qPCR testing. Performance analysis of the diagnostic antigen test showed the positive predictive value (PPV) to be 100% and negative predictive value (NPV) to be 98.10%, indicating that 1.90% of individuals with a negative result were, in fact, positive. If these data are extrapolated to the national level, where the mean daily number of antigen tests was 250,000 in April 2021, it points to over 4700 people per day being misinterpreted and posing a risk of virus shedding. While mean Ct values of the samples that were both antigen and RT-qPCR positive were about 20 (kit 1: 20.47 and 20.16 for *Sarbeco* *E* and *RdRP*, kit 2: 19.37 and 19.99 for *Sarbeco* *E* and *RdRP* and kit 3: 17.47 for *ORF1b*/*RdRP*), mean Ct values of the samples that were antigen-negative but RT-qPCR-positive were about 30 (kit 1: 30.67 and 30.00 for *Sarbeco* *E* and *RdRP*, kit 2: 29.86 and 31.01 for *Sarbeco* *E* and *RdRP* and kit 3: 27.47 for *ORF1b*/*RdRP*). It confirms the advantage of antigen test in detecting the most infectious individuals with a higher viral load. However, the reporting of Ct values is still a matter of ongoing debates and should not be conducted without normalisation to standardised controls of known concentration.

## 1. Introduction

Severe acute respiratory syndrome coronavirus 2 (SARS-CoV-2) belongs to the family *Coronaviridae*, genus *Betacoronavirus* and subgenus *Sarbecoviruses* [1]. The members of the *Coronaviridae* family are large RNA viruses, with enveloped viral particles ranging from 118 nm to 140 nm in diameter, bearing a single-stranded, positive RNA genome whose size ranges from 25 to 32 kb, coding for 16 non-structural and 4 structural proteins [2,3]. They infect a wide variety of host organisms, including birds, rodents, carnivores, bats, marine mammals, primates and, importantly, humans [4,5]. Up to now, seven coronaviruses have been linked to human diseases. Four of them (HCoV-HKU1, HCoV-OC43, HCoV-229E, HCoV-NL63) usually cause only the common cold or rhinitis with mild symptoms. The other three have been responsible for outbreaks of severe acute respiratory syndrome with significant morbidity and mortality: namely, SARS-CoV in 2002, in Guangdong, China, with 744 deaths out of 8098 infections; MERS-CoV in Saudi Arabia in 2012, with 866 deaths out of 2621 infections; and SARS-CoV-2, which emerged in Wuhan, China, at the end of 2019 and has since given rise to ongoing world-wide pandemic [3,4]. The first recorded cases were patients suffering from unusual viral pneumonia that, after initial ambiguities, was connected with a coronavirus of (at that time) unknown origin [6]. Later on, phylogenetic analysis suggested that bats might be the original host [7,8].

Only a few weeks later, on 30 January 2020, the WHO declared a global health emergency in response to COVID-19, the disease caused by SARS-CoV-2. According to WHO data, more than 128 million cases of COVID-19 and more than 2.8 million COVID-19-associated deaths were reported by 1 April 2021, when this article was completed [9]. Slovakia recorded its first case on 6 March 2020. From then to 1 April 2021, there were 362,489 confirmed cases of COVID-19 with 9790 deaths in Slovakia [10]. Despite early successes in containing the pandemic, the Slovak Republic became one of the countries with the highest daily COVID-19 death toll per 100,000 inhabitants during the first 3 months of 2021. The disease overran the health care system and brought it close to collapse. It also disrupted the economic and political situation. The first steps of the Slovak authorities to slow down the spread of the virus were the declaration of a state of emergency on 12 March 2020 and the introduction of strong anti-epidemic measures and border restrictions. Inspired by the example of South Korea [11], it started testing for the presence of the SARS-CoV-2, utilising the RT-qPCR method and introduced a mandatory quarantine for people coming from abroad. In spite of the measures taken by the Slovak Government and an increase in the volume of testing, the incidence of SARS-Cov-2-positive individuals began to grow rapidly in the autumn of 2020. Based on this situation, and in order to detect hotspots of infection, the Slovak Republic carried out a nationwide screening campaign using an antigen test (Standard Q COVID-19 Ag test, SD Biosensor) on 31 October and 1 November and subsequently on 8 November and 9 November 2020. The results and mathematical models of its effectivity were published by Pavelka et al. (2020) and Frnda and Durica (2020) [12,13]. Despite the achievement of a short-term reduction in cases, the epidemic situation rapidly deteriorated and became critical. The number of active cases and daily deaths continued to escalate [14] even after the introduction of more stringent measures. The tide did not turn until March 2021. This could be due to the expansion and predominance of new virus variants such as VOC-202012/1 (lineage B.1.1.7), which is thought to be associated with increased transmissibility, a higher reproduction number an increased risk of death [15,16].

Due to the need for healthcare workers to have daily direct contact with COVID-19 patients and despite using protective aids and strong hygienic measures, the number of SARS-Cov-2 positively tested doctors and nurses also increased, with direct negative impacts on the health care system. Under regulations issued by the Ministry of Health of the Slovak Republic, hospital employees are obliged to undergo testing using state-supplied antigen tests every second week. However, many questions were raised about the suitability of the selected testing method, especially regarding its use with large numbers of healthcare professionals and its effectiveness where individuals have low viral load [17,18,19]. 

At present, a wide range of methods is available for identifying SARS-CoV-2, including nucleic acid amplification testing (NAAT), serological testing, point-of-care testing, smartphone surveillance of infectious diseases, nanotechnology-based approaches, biosensors and next-generation sequencing (NGS) [20]. The main testing approaches involve detecting the virus itself (viral RNA or antigen) or detecting the human immune response to infection (antibodies or other biomarkers). While whole blood, serum or plasma can be used as a specimen for antibody-based immunoassays, upper or lower respiratory samples are used for antigen-based immunoassays and NAAT [21]. The primary NAAT method is RT-PCR, currently considered to be the gold standard of SARS-CoV-2 detection, and several targeted genomic regions (*ORF1b*, *N*, *RdRP*, *S* or *E* genes) have been used in the molecular diagnosis of virus RNA. The serological tests measuring binding antibodies (total immunoglobulins (Ig), IgG, IgM or IgA) make use of the enzyme-linked immunosorbent assay (ELISA), chemiluminescent immune-assays (CLIAs) for quantitative detection and lateral flow immunoassays (LFIA) for the rapid qualitative detection of SARS-CoV-2 [22]. Antigen testing relies on direct detection of SARS-CoV-2 proteins (spike or nucleocapsid) using LFIA. They are substantially less sensitive than NAAT, but on the other hand, they provide rapid, inexpensive and timely detection of the most infectious COVID-19 cases [23]. 

In this study, we analysed the state of coronavirus infections in Martin University Hospital using one rapid antigen test and three RT-qPCR assays. The City of Martin has nearly 55,000 inhabitants and occupies a central position both in Slovakia and Europe as a whole. It has an excellent hub hospital providing comprehensive healthcare to patients with a wide range of diagnoses, including COVID-19.

## 2. Results

### 2.1. SARS-CoV-2 Antigen Detection

**Figure 1:**
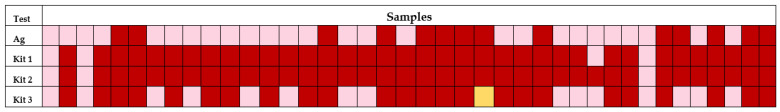
Consistency of positive cases by Ag—antigen test and three different RT-qPCR diagnostic kits, notes: dark red—positive, light red—negative, yellow—inconclusive result/invalid test (sample 826).

Out of 1231 samples, 14 were evaluated as positive for the presence of SARS-CoV-2 antigens (Figure 1). That extrapolated to a positivity of 1.14% among the hospital staff. Almost 90% of positive cases reported experiencing flu- or COVID-19-like symptoms at the time of testing.

### 2.2. SARS-CoV-2 RT-qPCR Testing

Out of 1227 collected naso/oropharyngeal swabs, 1223 produced valid RT-qPCR results. After the first analyses using the vDetect COVID-19 RT-qPCR diagnostic kit (MultiplexDX, Bratislava, Slovakia), we detected 23 positive samples (positivity 1.88%) and 17 with inconclusive results where there was a positive signal in the *Sarbeco gene E* but not in the *RdRP* gene. Such indefinite results are suggestive of a coronavirus infection other than SARS-CoV-2 or a processing error. To check this, all 40 samples (23 positive and 17 inconclusive) were re-isolated and re-analysed with the three RT-qPCR kits on the same day to avoid risks related to RNA freezing. The three tests, respectively returned positive results in 36, 37 and 24 cases, giving positivity rates for the samples amounting to 2.94%, 3.03% or 1.96%. The variability between the diagnostic kits is visualised in Figure 1. 

**Figure 2:**
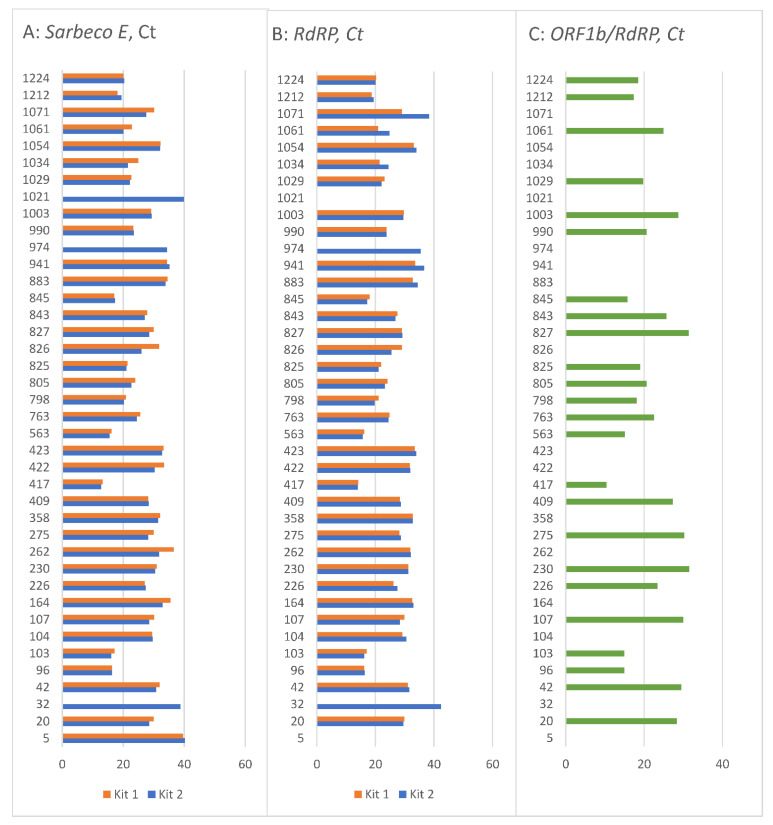
Ct values of selected 40 samples (**A**) Ct values of *Sarbeco E* gene by kit 1 and kit 2, (**B**) *RdRP* gene by kit 1 and kit 2, (**C**) *ORF1b* and *RdRP* by kit 3; note: no line = no Ct detected.

All the Ct values of the targeted genes in the 40 samples are shown in Figure 2. As can be seen, the same sample with the same pre-analytical processing can have different Ct values if another assay is used. Slight differences can be observed between kit 1 and kit 2 (Figure 2A,B), except for the samples 32, 974 and 1021. These three samples had either “no Ct” detected by kit 1 or values close to/above 40, measured by kit 2. Figure 2C shows the third testing kit, separately due to its different target genes, where more samples had “no Ct” values with a higher proportion of negative samples than the other two kits detected. 

**Figure 3:**
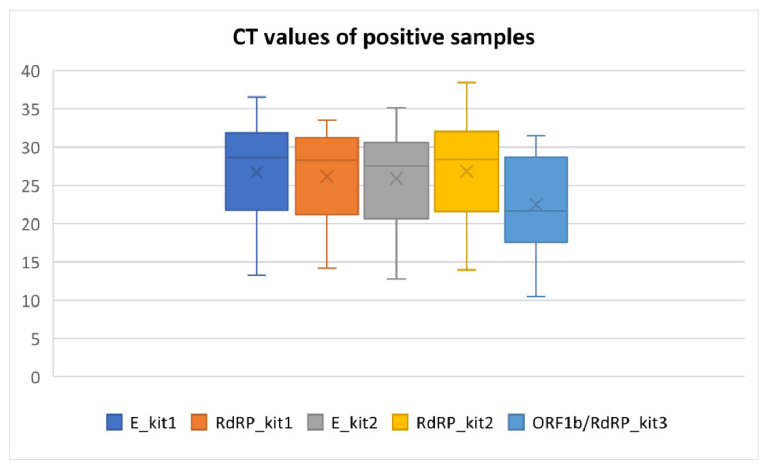
Box plots of measured Ct values of targeted genes by three different diagnostic kits (minimum, first quartile, mean, median, third quartile and maximum are depicted).

The mean Ct values and other descriptive parameters of RT-qPCR positive samples detected by three assays are listed in Table 1 and Figure 3. As these kits variate in the sensitivity, chemistry of reagents and gene targets, we provide only row data without statistical comparison between assays. More information about Ct values and their interpretation can be found in the Section 3 (Discussion). 

### 2.3. Antigen and RT-qPCR Testing

All 14 antigen-positive samples were confirmed in RT-qPCR tests using kit 1 and kit 2. Kit 3 failed to detect two samples with an inconclusive result, reporting no Ct either for targeted genes or *RNase P* control gene (Figure 1).

Moreover, RT-qPCR kit 1, 2 and 3 detected another 22, 23 and/or 12 positive cases respectively, among individuals where the antigen tests had been negative (Figure 1). 

Out of these three kits, we selected kit 2 (Generi Biotech, Hradec Králové, Czech Republic) to give the most reliable results, based on our routine laboratory testing experiences, stability of the reagents, clear guidelines for the sample processing and result interpretation, LOD specifications and inclusion of internal RT-PCR control. With this kit, we also passed external quality control for 100%. Therefore, we used data from kit 2 to calculate the sensitivity and specificity of the Standard Q COVID-19 Ag test. It was returned to be 37.84% and 100%. 

Analyses of the performance of the diagnostic antigen test showed the PPV 100% and NPV 98.10%, indicating all individuals with positive results were really positive, but 1.9% of individuals with negative results should have had a positive test result.

**Figure 4:**
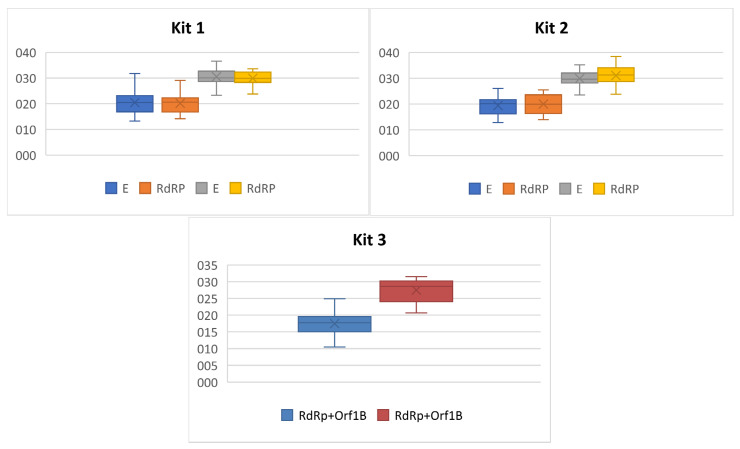
Box plots of SARS-CoV-2 targeted genes Ct values in RT-qPCR positive samples according to the antigen status.

Based on the Ag status and RT-qPCR testing, we had two groups: samples Ag and RT-qPCR positive and samples Ag negative but RT-qPCR positive. As seen in Figure 4 and Table 2, the mean Ct values of the first group is about 20, while the mean Ct values of those not detected by Ag testing were about 30. Roughly, it could be interpreted that the rapid antigen test is only able to detect samples with lower Ct values corresponding with higher viral load.

## 3. Discussion

**Figure 5:**
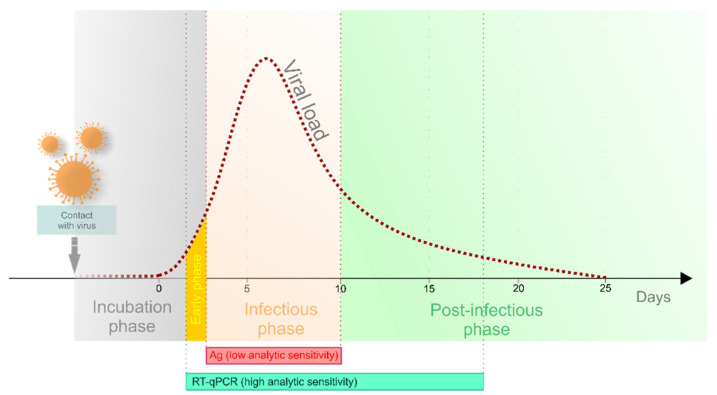
Analytic sensitivity of Ag and RT-qPCR tests, modified from Crozier et al. [29] with permission.

Several different tests have been developed for the diagnosis of COVID-19 since the outbreak of the pandemic. Each of them has specific analytic sensitivity, specificity and time limitation during the disease course (Figure 5) [24,25,26,27]. Neither antigen nor antibody detection is applicable for the purpose of early diagnosis. Antibody detection plays a role in serosurveys and can also serve as the proof of clearance from COVID-19 infection. At present, the method of RT-qPCR is considered the gold standard for the early diagnosis of SARS-CoV-2, providing the most accurate results during all disease phases [24,28], although various antigen tests are more widely used.

This study provides data-based confirmation of the expected differences between a rapid antigen test and RT-qPCR kits as well as between RT-qPCR assays. Our results have strong local significance in the Slovak Republic as the same rapid antigen tests have been widely used in the massive nationwide testing that commenced in fall 2020 [12,13]. From November 2020 to February 2021, specific institutions, factories and social service facilities were obliged to screen their employees regularly. The use of the tests became even more widespread at the beginning of February 2021, when the “COVID automat”—a set of uniform public health rules for the pandemic—was put into operation. At the highest level of alert then applying, periodic antigen-test screening became compulsory at intervals from 7 to 21 days. Regular testing was obligatory for most of the population until May 2021 [30].

### 3.1. Antigen Testing

In our study, we detected 14 positive samples (out of 1231) with the antigen test, representing 1.14% positivity among the hospital employees. All these results were confirmed by RT-qPCR kit 1 and kit 2. Kit 3 confirmed only 12 cases. In addition, the RT-qPCR tests detected another 22, 23 or 12 positive samples (this discrepancy might be caused by the different detection limit of the tests, which will be discussed later). Thus, up to 23 false-negative samples were identified and based on the new data; staff positivity was corrected to 3.03%. The negative predictive value (NPV) of the antigen test was calculated to be 98.10%. When extrapolated to the national level, more than 4700 people undergoing antigen tests each day might have been misdiagnosed as negative based on the mean daily total of 250,000 antigen tests in April 2021 [31]. The disadvantages of this low analytic sensitivity should therefore be precisely assessed, and consideration should be given to the question of whether they do not outweigh the advantages. However, after the analyses of Ct values, it can be seen that subjects that were both antigen and RT-qPCR positive had mean and median Ct values for *Sarbeco E* and *RdRP* genes amounting to about 20, whereas the antigen-negative but RT-qPCR-positive samples had these measures of centre close to 30 (details are in Table 2). The inability to detect samples with higher Ct values is in line with the results of Thommes et al. When investigating subjects with Ct values higher than 30, the antigen tests were positive in no more than 45% of such cases [17]. If the cycle threshold (Ct) values are taken as viral load indicators and prediction markers [32,33,34], it may be considered a strength of this antigen test (Standard Q COVID-19 Ag test, SD Biosensor) that it detects people at higher risk both on the personal level (risk of more severe disease) and on the population level (risk of viral shedding and transmission). Several studies reported significant associations between the Ct values at the time of diagnosis or hospital admission and COVID-19 disease severity, number of signs and symptoms, related death as well as sequelae presence several months after the disease [33,34,35,36]. 

On the other hand, the individuals with higher Ct are assumed to have a lower viral load at the time of sampling, and thus, they are considered to pose a lower risk of virus transmission [25]. It would be very useful to know the current health status in such cases when interpreting RT-PCR results. Patients who have overcome the disease and have decreased viral load or residual fragments resulting in a higher Ct value are of less concern than patients with the same Ct value who, on the contrary, have an early phase with increasing viral load potential [37]. Such individuals pose a threat because in the course of time, the viral load may exceed the critical level sufficient for effective viral shedding, and such carriers could inadvertently spread the disease despite the “favourable” outcome of the antigen test. Due to the technical limitations of the experiment, we did not perform follow up of the false-negative samples, which we considered to be a weak spot of our study. In our diagnostic practice, we had the opportunity to monitor the dynamic of Ct values in several patients, and as expected, the results showed a decrease in Ct values-thus increased viral load at the time of progressive development of the disease and vice versa, increased Ct values—thus decreased viral load—during the clearance of the disease (data not shown). Such observations highlight for us the importance of surveillance. Unfortunately, it is not a part of the standard diagnostic routine in Slovakia. 

In this study, no false positivity was detected from the antigen test. In clinical practice, we have encountered a few cases where RT-qPCR verification of a positive antigen test disproved the original result.

In our assessment of the accuracy of antigen testing, we calculated its diagnostic sensitivity and specificity to be 37.84% and 100% compared to RT-qPCR results from the second kit. Although the RT-qPCR methodology is accepted as the gold standard, no assay has 100% accuracy, with the problem more often being lower sensitivity and false negativity than false positivity [38]. Therefore, values of antigen diagnostic accuracy calculated in this way have limitations, are never exact and provide only an approximate estimate.

### 3.2. RT-qPCR and Ct Values

This study, besides the demonstration of antigen test unreliability, showed a non-negligible discrepancy between three different commercial RT-qPCR kits in their identification of 36, 37 and 24 positive cases, respectively. 

In our view, these differences could be caused primarily by differences in the limit of detection (LOD), declared to be 2 copies per reaction in kit 1 (vDetect COVID-19 RT-qPCR diagnostic kit, MultiplexDX, Bratislava, Slovakia), 2.13 copies/rxn for kit 2 (gb SARS-CoV-2 Multiplex, GENERI BIOTECH, Hradec Králové, Czech Republic) and 10 copies per reaction in kit 3 (Genvinset COVID-19 [E] kit, BDR Diagnostics, Zaragoza, Spain). As we expected based on the LOD, the mean Ct values of targeted genes were similar in the first and the second kits (Table 1). The third kit, requiring a higher quantity of the virus for reliable detection, based on the weakest LOD parameter, showed lower mean Ct values. It must be noted that this third test is a one-tube assay detecting both targeted genes in the FAM channel simultaneously, which could have an impact on Ct values. Considering the positivity rates, it appears that either kit 3 has lower sensitivity than kits 1 and 2, or the first and second kit have lower specificity than kit 3. From the molecular point of view, it seems that kits 1 and 2 were able to detect even just a few copies of the virus RNA. Whatever the epidemiology in terms of infectivity of the individual, risk of virus transmission and positive/negative status, as soon as the RNA is detected, the patient should be informed. Additionally, due to COVID-19’s character and the rapid spread of the virus, over-diagnosis due to higher sensitivity might be preferable to under-diagnosis because of selecting a kit with higher specificity. The inclusion of results for antibody detection would provide a comprehensive picture of the disease phase. 

These results contribute to the ongoing debates on whether to report Ct values or not, if it has individual utility in clinical decision-making or predicting prognosis and whether it realistically reflects viral load [33,37,39,40,41,42]. It is known that Ct values obtained by RT-qPCR can vary. Payne et al. wrote that over 60% of errors occur in the pre-analytical phase of any diagnostic process [43]. In the SARS-CoV-2 context, this phase includes the type of sample, the method of specimen collection, transport medium composition, storage conditions and time from sample collection to the RNA isolation [43,44]. The RNA extraction method and the RT-qPCR assay has also significant impact on Ct values, as demonstrated by this study [39,40,45,46]. The selection of the optimal kit is a complex process as the quality of RT-qPCR assays depends on multiple factors. Parameters of great importance include LOD, sensitivity and specificity, avoidance of cross-reactivity with other pathogens, fluorescence parameters and primer quality. Additionally, the inclusion of the human control probe and internal control are vital for the sample quality control and RT-qPCR control. Another key consideration is the stability of the chemical components through repeated thawing and refreezing and the ability to detect not only fresh but also frozen RNA. Based on our evaluation of these factors, we selected the kit from GENERI BIOTECH as the best, meeting the needs of our day-to-day laboratory work. The version of the first kit by MultiplexDX had a perfect LOD but was less satisfactory in its chemical components and Ct setup. As this company has optimised its assays, another version might have increased quality, similar to their well-working multiplex assay. Kit development and optimisation are ongoing processes all over the world and are ever more urgent as we face the rise and spread of new virus variants. Many different, upgraded commercial kits are available for the diagnosis of patients with SARS-CoV-2 [47,48,49,50,51]. As they significantly differ in the above-mentioned parameters, reporting row Ct values is very questionable, and Ct values cannot be directly compared between assays. We showed that one sample can have different Ct values analysed by three assays. On the other side, identical Ct from different kits could mean distinct infectivity. The Ct value of a more sensitive kit represents a lower viral load compared to the same Ct of a less sensitive kit which needs thousands of more virus copies to reach the same Ct. Additionally, as discussed above, the identical Ct detected by the same assay has distinct infectivity potential if an early or late phase is detected. Without clinical data, the RT-PCR test cannot tell the disease phase [44]. Discrepancies between Ct values from different assays could be solved by normalisation by standard curve allowing Ct translation into copies/mL [52,53,54]. This serial dilution normalisation and viral load calculation are missing in our study and routine testing as, according to national guidelines, is not required. If all laboratories adopt the normalisation, results of RT-PCR could also have quantitative character informing more precisely about viral load. Additionally, in combination with actual health status and specific guidelines for pre-analytical processing, the RT-PCR testing would be a real “gold standard” technique for SARS-CoV-2 detection. To ensure it, standardised global protocols are needed in this pandemic situation. International authorities or pan-region reference laboratories for SARS-CoV-2 detection could take this role and uniform the testing, clearly state the criteria for diagnostic assays as the CE IVD mark does not automatically guarantee high kit quality and reliability and provide guidelines for Ct translation and virus copies reporting. It is probable that the current pandemic is not the last one. Given the unprecedented growth of the human population, climate change and continuing encroachment into uninhabited areas suitable for wildlife, it seems impossible to prevent interactions between humans and animals, through which more zoonotic viruses such as coronaviruses enter society [6].

## 4. Materials and Methods

### 4.1. Subjects and Sampling

**Figure 6:**
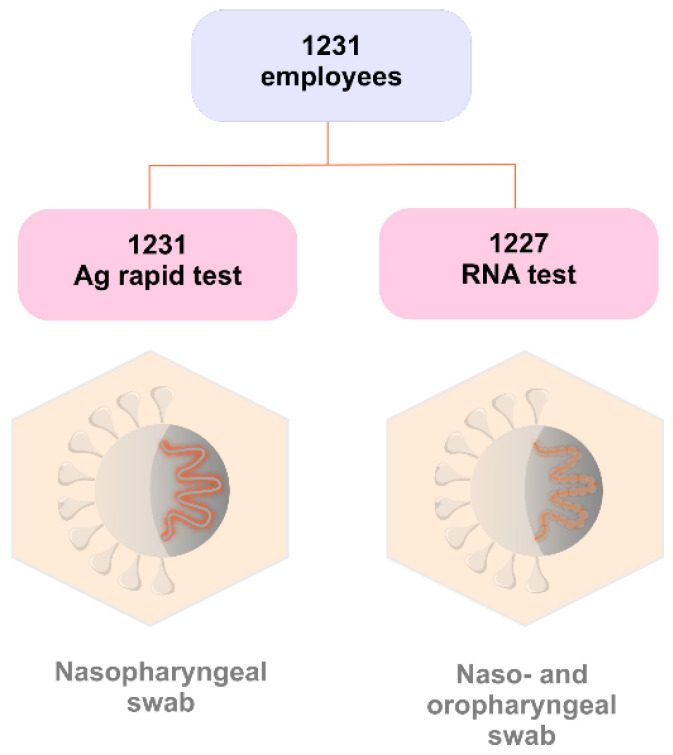
The study design and specimen collection: 1231 nasopharyngeal swabs for rapid detection of nucleocapsid protein and 1227 naso/oropharyngeal swabs for detection of SARS-CoV-2 RNA.

1231 out of 2408 employees of the Martin University Hospital were enrolled in this study for SARS-CoV-2 antigen and RNA detection. Specimens were collected from subjects via naso/oropharyngeal swabs at the same time on 3 and 4 December 2020 (Figure 6). The study was designed in line with the ethical principles of Helsinki declaration and was approved by the Ethics committee (No. EK45/2020). All participants of the study signed informed consent. 

### 4.2. Antigen Testing

The rapid chromatographic immunoassay Standard Q COVID-19 Ag test (SD Biosensor) was used for rapid qualitative detection of SARS-CoV-2 antigens present in the human nasopharynx. This test detects nucleocapsid protein of SARS-CoV-2 by its reaction with mouse monoclonal anti-SARS-CoV-2 antibodies conjugated with colour particles in the device. Testing was performed according to the manual: the nasopharyngeal swab from the nasal cavity was inserted into the extraction buffer, stirred at least 5 times and removed while squeezing the side of the tube. Three drops of extracted specimen were applied to the specimen well of the test device and read after 15–30 min. The samples were evaluated as positive if they had two purple-coloured bands, marking both the C (control) line and the T (test) line. Samples with a coloured band on only the C (control) line had negative status.

### 4.3. RNA Isolation

Total RNA was extracted using a Quick-RNA™ Viral 96 kit (Zymo Research, Waltham, MA, USA, cat#R1041), according to the manufacturers’ guidelines and calculated for increased volume of 200 μL of the sample. Briefly, 200 μL of DNA/RNA Shield^TM^ 2× concentrate was added into 200 μL of viral transport medium containing the nasopharyngeal swab. Then, the mixture was combined with Viral RNA Buffer and transferred into separate wells in a Zymo-Spin™ I-96 Plate mounted on a Collection plate. Washing and centrifugation steps were performed, and the viral RNA was eluted with 25 μL of DNase/RNase-Free Water into an elution plate and subsequently used for RT-qPCR detection.

### 4.4. RT-qPCR Testing by Three Different CE IVD Kits 

All 1231 naso- and oro-pharyngeal swab samples were first analysed using kit 1: vDetect COVID-19 RT-qPCR diagnostic kit (MultiplexDX, Bratislava, Slovakia) consisted of SARS-CoV-2 viral RNA testing using one-step RT-qPCR. It targets the novel coronavirus envelope (*E*) gene and human *RNase P* as the internal control. Positive results were subsequently confirmed by viral RNA-dependent RNA polymerase (*RdRP*) gene. Each reaction contained a set of primers and hydrolysis dual labeled TaqMan^®^ probes (targeting *E* or *RdRP* or *RNase P*) mixed with the 2× Brilliant III Ultra-Fast QRT-PCR Master Mix, RT/RNase Block, 100 mM DTT and PCR water in total volume of 15 μL and 5 μL of RNA extracted from nasopharyngeal swab. The thermal cycling conditions were: reverse transcription at 50 °C for 30 min, initial denaturation at 95 °C for 3 min and 45 cycles with denaturation at 95 °C for 5 s and annealing/extension at 60 °C for 20 s. A positive result was interpreted if both viral genes *E* and *RdRP* and internal control human *RNase P* amplification curves crossed the threshold line within 40 cycles. As this test gave several inconclusive results, all samples with at least one signal in either the *E* or *RdRP gene* were re-isolated and re-analysed by this and other two kits on the same day to avoid RNA freezing as we found that frozen RNA did not work well with the RdRP assay from kit 1 (Table 3). 

Kit 2: gb SARS-CoV-2 Multiplex (GENERI BIOTECH s.r.o., Hradec Králové, Czech Republic) reactions contained the Master Mix OneStep Multi and the Assay CoV-2 E-RdRP or B2M in a total volume of 15 μL and 5 μL of extracted RNA. Thermal cycling conditions were: reverse transcription at 42 °C for 30 min, initial denaturation at 95 °C for 3 min and 50 cycles with denaturation 95 °C for 10 s and annealing/extension at 60 °C for 30 s with fluorescence acquisition for FAM, HEX and Cy5 channels. The samples were evaluated as positive if a fluorescence signal was present in the FAM channel specific for the *E* and *B2M* genes, and in the HEX channel specific for *RdRP*. Positive external control was read in the Cy5 channel.

The Genvinset COVID-19 [E] kit (BDR Diagnostics, Zaragoza, Spain) uses Stabilized Amplification Technology-Nucleic Acid Testing (STAT-NAT^®^) technology. Lyophilised Master Mixes were reconstituted in 15 μL of enclosed buffer to which 10 μL of extracted RNA were added. The recommended thermal cycling conditions were reverse transcription at 50 °C for 10 min, initial denaturation at 95 °C for 2 min, 10 cycles without fluorescence detection at 95 °C for 15 s and at 58 °C for 30 s and following 35 cycles with denaturation 95 °C for 15 s and annealing/extension at 60 °C for 30 s with fluorescence acquisition for the FAM and HEX channels. The sample was evaluated as positive if the fluorescent signal was present in the FAM channel for two target regions of *ORF1b* and *RdRP* genes and present or absent in the HEX channel for human *RNase P* gene as an internal control for amplification. All negative samples presented a signal in the HEX channel. 

All qRT-PCR analyses were performed on qTOWER^3^ (Analytik Jena GmbH, Jena, Germany). The RT-qPCR results were analysed using the instrument software (qPCRsoft 4.0) with a constant threshold line setting.

### 4.5. Statistical Analyses

The IBM SPSS statistical program v.21 was used to calculate descriptive statistics. The paired T-test was used to compare the mean Ct values between diagnostic tests. A non-parametric Wilcoxon test for independent samples was used to compare Ct values between two diagnostic groups (Ag-positive and Ag-negative samples, both RT-qPCR positive).

The sensitivity of the rapid antigen test was calculated as true positive/(true positive + false negative) × 100, and the specificity was calculated as true negative/(true negative + false positive) × 100.

We also calculated the performance of the antigen diagnostic test by the positive predictive value (PPV) and negative predictive values (NPV) as follows: PPV = true positive/(true positive + false positive) and NPV = true negative/(true negative + false negative).

## 5. Conclusions

To conclude the results of this study by summing up our experience and expectations, the main outcomes could be defined as:

- The results confirmed that the Standard Q COVID-19 Ag test (SD Biosensor) has an advantage in its ability to detect people with lower Ct values, who can be assumed to have higher viral load and thus pose the highest risk of virus transmission.
- The relatively high number of false-negative results from the antigen test increases the risk of silent virus shedding in the population. If used in mass testing, thousands of individuals could receive incorrect results and proceed to spread the virus, especially if tested at an early phase of viral infection.
- The antigen test is therefore time-limited, and its utilisation in blanket population screening should be re-considered given that the benefit vs. risk ratio is questionable.
- The sensitivity and specificity of an antigen test compared to the gold standard RT-qPCR testing is not accurate unless RT-qPCR sensitivity limits are included in the algorithm.
- Providing row Ct values is not sufficient. We proved that one sample can have variable Ct values if different assays are used. Moreover, the same Ct value of kits with different sensitivity represent a discrepant amount of virus copies (even in the thousands). Additionally, identical Ct detected by one kit can have different infectivity aspects depending on the disease phase.
- The algorithm for RT-PCR testing should also calculate actual health status, and if an early phase is suspected, retesting should be considered in the cases when Ct was detected above the threshold resulting in a negative result.
- Clear guidelines for pre-analytical, analytical and post-analytical steps, including chemistry quality, are needed to uniform the testing.
- Normalisation of Ct values by standard dilution curve, counting virus copies could allow inter-kit comparison between laboratories and epidemiological monitoring.

## Figures and Tables

**Table 1:** Descriptive statistics of Ct values in positive samples.

	Targeted Genes				
	Kit 1		Kit 2		Kit 3
	*E*	*RdRP*	*E*	*RdRP*	*ORF1b*/*RdRP*
*n*	36	36	37	37	24
Median	28.64	28.28	27.51	28.36	21.65
Mean	26.70	26.17	25.89	26.84	22.47
SD	6.30	5.81	6.03	6.53	6.21
Min	13.24	14.16	12.80	13.96	10.45
Max	36.55	33.54	35.12	38.42	31.50

Notes: *n*—number of samples where the Ct value was detected, SD—standard deviation, Kit 1 (MultiplexDX), Kit 2—Generi Biotech, Kit 3—BDR Diagnostics.

**Table 2:** Descriptive statistics of Ct values of RT-qPCR samples according to Ag status.

	Kit 1				Kit 2				Kit 3	
	Ag +		Ag −		Ag +		Ag −		Ag +	Ag −
	*E*	*RdRP*	*E*	*RdRP*	*E*	*RdRP*	*E*	*RdRP*	*ORF1b*/*RdRP*	*ORF1b*/*RdRP*
*n*	14	14	22	22	14	14	23	23	12	12
median	20.48	20.61	30.14	29.92	20.10	19.90	29.62	31.20	17.77	28.59
mean	20.47	20.16	30.67	30.00	19.37	19.99	29.86	31.01	17.47	27.47
SD	4.71	3.86	3.19	2.77	3.46	3.73	2.97	3.68	3.64	3.59
min	13.24	14.16	23.31	23.76	12.80	13.96	23.52	23.74	10.45	20.70
max	31.80	29.03	36.55	33.54	26.00	25.45	35.12	38.42	24.92	31.50

**Table 3:** Details of the RT-qPCR kits used in this study.

	Kit 1	Kit 2	Kit 3
Kit Name	vDetect COVID-19 RT-qPCR Diagnostic Kit	gb SARS-CoV-2 Multiplex	Genvinset COVID-19 [E] kit
Company	MultiplexDX, Slovak republic	GENERI BIOTECH s.r.o., Czech republic	BDR Diagnostics, Spain
Targeted genes	*E*, *RdRP*	*E*, *RdRP*	*ORF1b* + *RdRP*
Control gene	*RNase P*	*B2M*	*RNase P*

Notes: Gene *E*—gene encoding the small membrane envelope protein of the SARS-CoV-2 virus, target region general for *Sarbecoviruses* in kit 2 and specific for SARS-CoV-2 in kit 1; Gene *RdRP*—gene encoding the RNA-dependent RNA polymerase of SARS-CoV-2 virus; *RNase P*—gene encoding human nuclear ribonuclease P; *B2M*—gene encoding human beta-2-microglobulin; *ORF1B*—gene encoding open-reading frame 1b of the SARS-CoV-2 virus.

## Data Availability

The data presented in this study are available on request from the corresponding author.

## Abbreviations

*B2M*	the gene encoding human beta-2-microglobulin
COVID-19	coronavirus disease 2019, the official name for the disease caused by the virus
Ct	cycle threshold
*E gene*	the gene encoding the small membrane envelope protein of the SARS-CoV-2 virus
LOD	limit of detection
NPV	negative predictive value
*ORF1B*	gene encoding open-reading frame 1b of the SARS-CoV-2 virus
PPV	positive predictive value
*RdRP gene*	the gene encoding the RNA-dependent RNA polymerase of the SARS-CoV-2 virus
*RNase P*	the gene encoding human nuclear ribonuclease P
RT-qPCR	quantitative reverse transcription PCR
SARS-CoV-2	severe acute respiratory syndrome coronavirus 2, the official name for the virus causing the disease
